# Microsecond dark-exciton valley polarization memory in two-dimensional heterostructures

**DOI:** 10.1038/s41467-018-03174-3

**Published:** 2018-02-21

**Authors:** Chongyun Jiang, Weigao Xu, Abdullah Rasmita, Zumeng Huang, Ke Li, Qihua Xiong, Wei-bo Gao

**Affiliations:** 1 Division of Physics and Applied Physics, School of Physical and Mathematical Sciences, Nanyang Technological University, Singapore, 637371 Singapore; 2 NOVITAS, Nanoelectronics Center of Excellence, School of Electrical and Electronic Engineering, Nanyang Technological University, Singapore, 639798 Singapore; 3 MajuLab, CNRS-Université de Nice-NUS-NTU International Joint Research Unit UMI 3654, Singapore, 637371 Singapore; 4 The Photonics Institute and Centre for Disruptive Photonic Technologies, Nanyang Technological University, 637371 Singapore, Singapore

## Abstract

Transition metal dichalcogenides have valley degree of freedom, which features optical selection rule and spin-valley locking, making them promising for valleytronics devices and quantum computation. For either application, a long valley polarization lifetime is crucial. Previous results showed that it is around picosecond in monolayer excitons, nanosecond for local excitons and tens of nanosecond for interlayer excitons. Here we show that the dark excitons in two-dimensional heterostructures provide a microsecond valley polarization memory thanks to the magnetic field induced suppression of valley mixing. The lifetime of the dark excitons shows magnetic field and temperature dependence. The long lifetime and valley polarization lifetime of the dark exciton in two-dimensional heterostructures make them promising for long-distance exciton transport and macroscopic quantum state generations.

## Abstract (web summary)

Owing to their valley degree of freedom, atomically thin transition metal dichalcogenides show promise for valleytronics. Here, the authors leverage the dark excitons of two-dimensional van der Waals heterostructures to demonstrate a microsecond valley polarization memory effect.

## Introduction

Reassembled layered van der Waals heterostructures have revealed new phenomena beyond single material layers^1^. In particular, when two different monolayer transition metal dichalcogenides (TMDs) are properly aligned, the electrons can be confined in one layer while the holes are confined in the other layer^1–8^. Because the electron and hole wavefunctions in the two layers only have small overlap, excitons can have a longer lifetime of several nanoseconds, as compared to around picoseconds for excitons in monolayer. Such exciton is known as indirect exciton or interlayer exciton^2,8^. A long exciton lifetime is crucial for the generation of high-temperature macroscopically ordered exciton state, which forms the basis of a series of fundamental physics phenomena such as superfluidity^9^ and Bose–Einstein condensation^10–12^. Moreover, longer survival of excitons means longer distance of exciton transport, which is useful for excitonic devices^13,14^.

In another perspective, similar to monolayer TMDs, the properly aligned two-dimensional (2D) heterostructure has indirect exciton with valley degree of freedom. Valleys (K and K′) are located at the band edges in the corners of the hexagonal Brillouin zone^15,16^. The spins show opposite signs in the two valleys at the same energy which corresponds to spin-valley locking^15,16^. Moreover, these two valleys have opposite Berry curvature leading to different optical selection rules in each valley. By using a circularly polarized optical pumping and observing the locking between the output photon chirality and the valleys, the phenomena of valley polarization has been observed^17–21^. These unique properties put the valley degree of freedom as a possible candidate for opto-electronics and quantum computation with 2D material^22–24^. Leading to its application, a long valley polarization lifetime is a prerequisite and extensive efforts have been put towards particles and quasi-particles with longer lifetime in these ultra-thin systems. Previous lifetime measurement reveals that direct exciton lifetime is in the order of picosecond^25–27^, limiting its application to some extent. Localized excitons can have a lifetime of around nanosecond^28,29^ and indirect exciton can have ~100 ns lifetime^30,31^ and tens of nanosecond valley polarization lifetime^8^. We note that recent work also shows single charge carrier can have microsecond lifetime^32^. On the other hand, experimental evidence shows the existence of dark excitons, lying tens of millielectronvolts below the bright exciton in WSe_2_^33^. Their decay time is measured to be nanoseconds in monolayer TMD^34^.

Here we report that, the dark excitons in 2D heterostructures can survive for microsecond timescale. With magnetic field suppressed valley mixing, they serve as a microsecond valley polarization memory for indirect excitons. This is two orders longer than the case without an applied magnetic field.

## Results

### Experimental observation of interlayer excitons

**Fig. 1:**
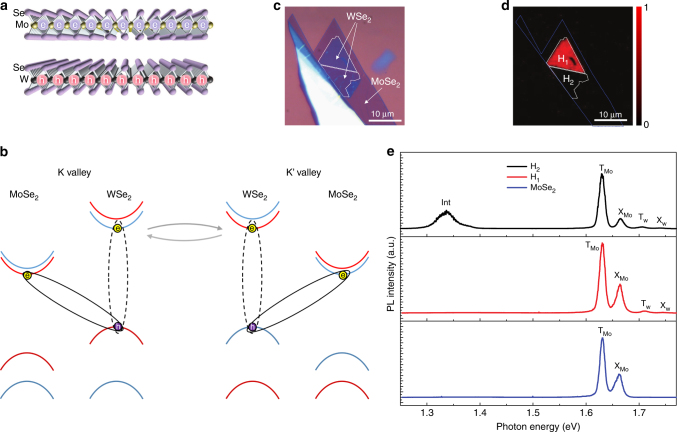
Sample characterization. **a** MoSe_2_ and WSe_2_ form a 2D heterostructures, where electrons are confined in one layer and holes are confined in the other layer. **b** Schematic of the interlayer exciton and dark exciton. The interlayer excitons are illustrated as solid black ellipses. The dark excitons are represented by the dashed ellipses. Red (blue) curves denote spin-up (spin-down) in the conduction and valence bands while the gray arrowed curves denote the dark exciton valley scattering. **c** Optical microscope image of the MoSe_2_/WSe_2_ heterostructure. Blue dashed line shows the area of MoSe_2_. White dashed lines show the region of heterostructure, which is separated into two areas labeled as H_1_ and H_2_ in **d**. **d** Fluorescence image taken with a color camera under white light excitation. It shows a bright (H_1_) and dark (H_2_) state in the two different place of heterostructure. **e** Photoluminescence in the monolayer MoSe_2_, heterostructure H_1_ region, and heterostructure H_2_ region under a 633 nm laser excitation. Peaks on MoSe_2_ are attributed to exciton (X_Mo_) and trion (T_Mo_) of MoSe_2_. In H_1_ area, another two weak peaks appear and are labeled as exciton (X_W_) and trion (T_W_) from WSe_2_. In H_2_ area, another peak around 1.34 eV emerges, which is attributed to interlayer exciton and labeled as Int

The schematics and the optical spectroscopy result of the MoSe_2_/WSe_2_ heterostructure on SiO_2_/Si substrate are shown in Fig. 1. In such heterostructures, electrons tend to go to the conduction band of MoSe_2_ and holes are confined in the valence band of WSe_2_, forming the indirect excitons (Fig. 1a). Our samples are prepared via a mechanical exfoliation and aligned-transfer method^7^. In this sample, the MoSe_2_ monolayer is stacked on top of the WSe_2_ monolayer in AA-stacking style (see Supplementary Note 4 and Supplementary Fig. 5 for the second harmonic generation experiment). The detail of the sample preparation can be found in the Methods section. A fluorescence image of the heterostructure was taken with a color camera under white light excitation (Fig. 1d). It is found that the heterostructure consists of two areas: dark area with low intensity luminescence (labeled as H_1_) and bright area with high intensity luminescence (labeled as H_2_). The photoluminescence (PL) of these two areas as well as the MoSe_2_ region under 633 nm continuous-wave (CW) laser excitation is shown in Fig. 1e. As can be seen from this figure, the interlayer exciton emission of ~1.34 eV emerges for the dark area H_2_. The interlayer exciton emission intensity is comparable to the intralayer exciton and the trion emission of MoSe_2_. The interlayer emission is missing for the H_1_ region where higher intensity of intralayer emission is observed. This can be attributed to the weak coupling between the two layers in this H_1_ region^7^. In the following measurements, we focused on the interlayer exciton emission in H_2_ area. An 850-nm long pass filter is used in the PL collection to filter out the contribution of other emission type.

### Valley polarization with CW laser

**Fig. 2:**
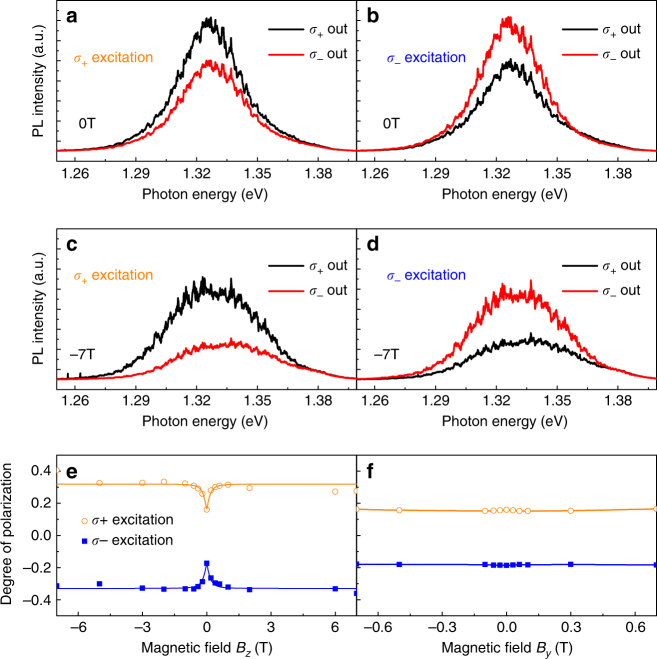
Valley polarization with CW laser excitation. **a**, **b** Valley polarization at 0 T. Right and left circularly polarized light are labeled as *σ*_+_ and *σ*_−_. Under *σ*_+_ laser excitation, *σ*_+_ PL output component is more than *σ*_−_ and vice versa for *σ*_−_ excitation. This shows evidence of valley polarization. **c**,** d** Valley polarization at −7 T. Valley polarization is enhanced by applying magnetic field perpendicular to the sample surface. **e** The valley polarization degree as a function of applied magnetic field in the *z* direction. The solid line is the fitting result following equation $P^j = P_0^j \pm P_1^j\left( {1 - \frac{1}{{r^2 + r\sqrt {1 + r^2} + 1}}} \right)$, $r = \left| B \right|{\mathrm{/}}\alpha$ where *j* indicates the excitation polarization, $P_0^j$ is the residual degree of polarization at 0 T due to the valley polarization, $P_1^j$ is the saturation level of degree of polarization, and *α* represents the intervalley scattering between the dark exciton. **f**, The valley polarization degree as a function of applied magnetic field in the *y* direction with *B*_*z*_ = 0T

We firstly carried out the measurement of valley polarization with a CW pump laser. The results are shown in Fig. 2. In order to increase the count of the interlayer exciton, an excitation laser with wavelength of 1.708 eV is used. This corresponds to the resonant excitation of the WSe_2_ charged exciton. The polarization states of the excitation and detection are set to the circular polarization *σ*_+_ or *σ*_−_, and the degree of circular polarization is extracted from these four polarization combinations. Please note that the emission polarization will have a small distortion away from circular polarization due to the existence of the Moire pattern^35–38^. However, circular polarization still acts as a good approximation for studying the dynamics of valley polarization here. The PL emission of different configurations at 0 T are shown in Fig. 2a, b. It is observed that the PL intensity of the co-polarization is always larger than that of the cross-polarization, corresponding to valley polarization. Next, we apply a magnetic field of −7 T perpendicular to the sample surface (out-of-plane direction, *B*_*z*_). The results are shown in Fig. 2c, d. As can be seen from these figures, the emission difference between the case with co-polarization and cross-polarization excitation gets larger at *B*_*z*_ = −7 T compared to 0 T.

To quantify this difference, we measured the degree of polarization as a function of magnetic field in both *B*_*z*_ (Faraday geometry) and *B*_*y*_ directions (parallel to sample surface, Voigt geometry). Here we define the degree of polarization as $P^j = \frac{{I_{\sigma _ + }^j - I_{\sigma _ - }^j}}{{I_{\sigma _ + }^j + I_{\sigma _ - }^j}}$, where $I_{\sigma _ + }^j$ $\left( {I_{\sigma _ - }^j} \right)$ is the *σ*_+_ (*σ*_−_) polarized PL intensity when excited with *j* polarization. The degree of polarization pumped by *σ*_+_ and *σ*_−_ excitation versus magnetic field in the Faraday and Voigt geometry are shown in Fig. 2e, f. We can observe a dip of the degree of polarization at low magnetic field around 0 T in Faraday geometry. The valley polarization is around 17% at 0 T and quickly increases to ~35% at ~1 T. For Voigt geometry, the degree of polarization does not show any dependence on the magnetic field.

Regarding the Faraday geometry, our observation of increased valley polarization near 0 T is in line with the report in ref. ^39^ where it is attributed to the suppression of intervalley electron–hole exchange interaction. Similar with traditional semiconductor quantum wells and quantum dots, the valley depolarization in 2D material is caused by electron–hole exchange interaction^40–42^. The larger binding energy of excitons in monolayer TMDs further enhances such interaction, leading to valley depolarization and short valley polarization lifetime. The intervalley scattering can be understood in term of in-plane depolarizing field^40^. Hence, by increasing the magnitude of out-of-plane magnetic field, the valley depolarization can be suppressed^28,41^. This model can also explain why the degree of polarization saturates at high magnetic field. This saturation has also been observed in WSe_2_ exciton system^43^. The difference is that, unlike the bright exciton case reported there, the interlayer exciton shows a non-linear magnetic-dependence of valley polarization. Following this, we fit the degree of polarization with equation   $P^j = P_0^j \pm P_1^j\left( {1 - \frac{1}{{r^2 + r\sqrt {1 + r^2} + 1}}} \right)$, $r = \left| B \right|{\mathrm{/}}\alpha$ where *j* indicates the excitation polarization, $P_0^j$ is the residual degree of polarization at 0 T, $P_1^j$ is the saturation level of degree of polarization, and *α* represents the intervalley scattering term between the dark excitons where the dark exciton refers to the WSe_2_ dark exciton as illustrated in Fig. 1b. The experimental data fits very well with this model within the magnetic field range of our experiment.

The exchange interaction-based argument can also be used to explain why the degree of polarization does not show any magnetic field dependence under the Voigt geometry. In order to discuss this, it should be noted that the 2D material can be seen as an atomic-thin quantum well. It is known that the effect of in-plane magnetic field to the exchange interaction is proportional to the quantum well thickness^44^. Hence, the exchange interaction is practically independent of the magnetic field under the Voigt geometry which result in the magnetic field-independent degree of polarization.

### Time-resolved experiment with pulsed laser excitation

**Fig. 3:**
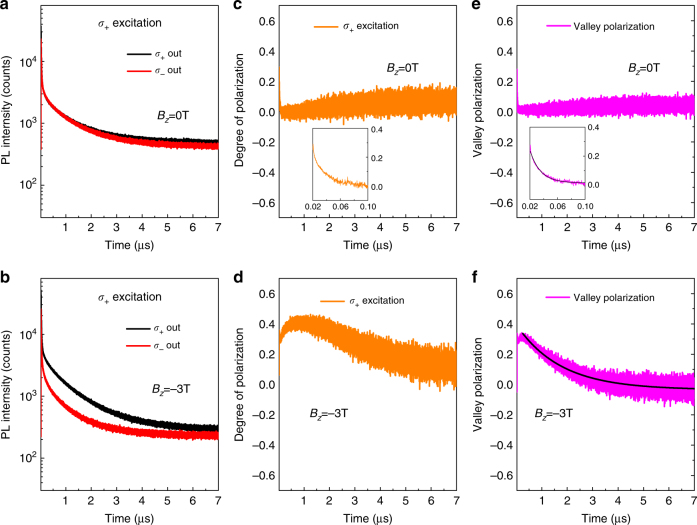
Time-resolved investigation of polarization in magnetic field. **a**,** b** Time-resolved PL of *σ*_+_-output and *σ*_−_-output pumped by a *σ*_+_-polarized pulsed laser in Faraday geometry at *B*_*z*_ = 0 T (**a**) and *B*_*z*_ = −3 T (**b**). **c**,** d** Degree of polarization extracted from *σ*_+_ excitation PL at *B*_*z*_ = 0 T (**c**) and *B*_*z*_ = −3 T (**d**). **e**, **f** Valley polarization calculated from *σ*_+_ excitation and *σ*_−_-excitation PL. The *σ*_−_ excitation PL data is in the Supplementary Figs. 1 and 2. At *B*_*z*_ = 0 T, the degree of polarization disappears quickly and hardly seen after 50 ns and valley polarization has a decay time of 15 ± 0.3 ns. At *B*_*z*_ = −3 T, the difference between *σ*_+_ output and *σ*_−_ output is clearly seen even at 200 ns. The valley polarization at *B*_*z*_ = −3 T has a decay time of 1.745 ± 0.007 μs

To understand the dynamics of the interlayer exciton emission, we carried out time-resolved PL experiment with pulsed laser excitation. The laser has a repetition period of 8 μs and has the same wavelength as the one used in the CW experiment. The left panels of the Fig. 3a, b show the decay of the PL emission pumped by *σ*_+_ excitation at 0 T and −3 T, respectively. The middle panels show the calculated degree of polarization. We can see that, the degree of polarization $P^{\sigma _ + }$ decays quickly to zero at 0 T, while it has an extra slow decay component and remains above 0.2 for up to 2.5 μs at −3 T. To quantify it, here we address two different types of degree of polarization: valley polarization and PL polarization. The former depends on the polarization state of the excitation, i.e. copolarization and cross-polarization give different PL intensity. It can be calculated as $P_{{\mathrm{val}}} = \frac{{P^{\sigma _ + } - P^{\sigma _ - }}}{2}$. The latter one solely depends on the polarization of the PL emission and it does not depend on the excitation. It can be calculated as the average of the individual degree of polarization pumped by *σ*_+_ or *σ*_−_ excitation: $P_{{\mathrm{PL}}} = \frac{{P^{\sigma _ + } + P^{\sigma _ - }}}{2}$. Figure 3e, f shows the decay of valley polarization *P*_val_ at 0 and −3 T. valley polarization at 0 T has a decay time of 15 ± 0.3 ns, while it has a decay time of 1.745 ± 0.007 μs at −3 T. More detailed PL data, valley polarization and PL polarization at −3, 0 and 3 T are provided in Supplementary Figs. 1 and 2. More analysis can be found in Supplementary Note 1.

### Microscopic mechanism of long lifetime and valley polarization lifetime for interlayer excitons

**Fig. 4:**
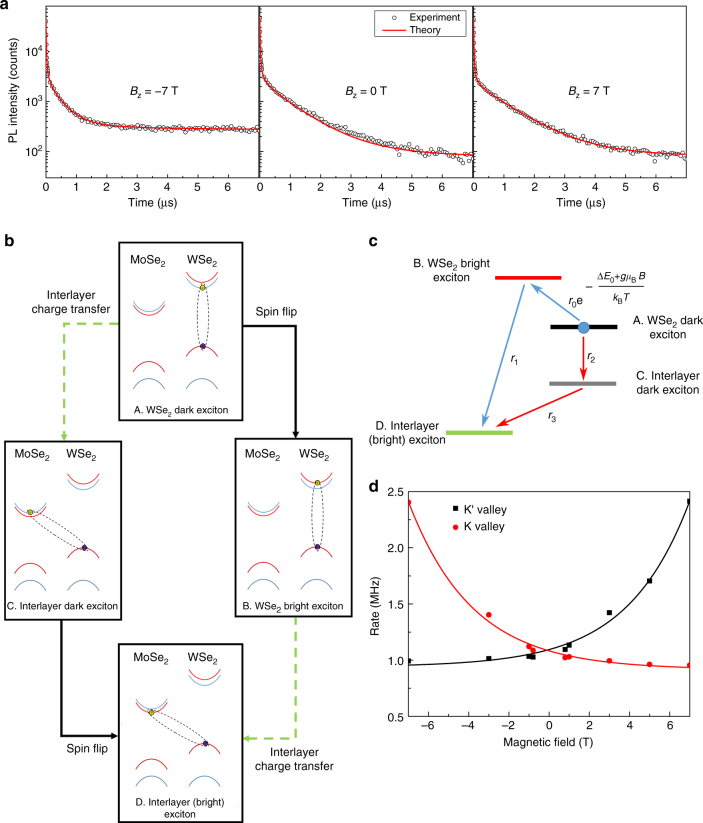
Theoretical model and experimental data fitting. **a** Sample of the experimental data (temperature, *T* = 2.3 K) and the fitting to the theoretical model. The data is obtained using *σ*_−_ polarized pulsed excitation and *σ*_−_ polarized PL detection. Semi-log plot is used. **b**, **c** The two conversion pathways from the WSe_2_ dark exciton to the interlayer exciton. In the first pathway (A-B-D), the spin flip happens before the interlayer charge transfer while in the second one (A-C-D), the interlayer charge transfer happens before the spin flip. The conversion rate in the first path has an exponential magnetic field dependence while it is constant for the second path. **d** Scattering rate from dark exciton to interlayer exciton versus magnetic field. The dark-to-interlayer scattering rates in K valley (*σ*_−_ PL) and K′ valley (*σ*_+_ PL) are plotted against magnetic field and fitted using exponential function

Below we analyze the origin of the long exciton lifetime and valley polarization lifetime. To this end, the experimental result for *B* = −7, 0, 7 T at low temperature (*T* = 2.3 K) in the case of *σ*_−_ polarization excitation and *σ*_−_ polarized PL detection are shown in Fig. 4a. First we consider the case at high magnetic field. The decay has both the slow and fast decay components. The slow decay in the order of *τ*_1_ ~ 1 μs suggests the dark exciton involvements, which will be further confirmed by the magnetic field dependence measurement shown below. The fast decay  part includes two parts, one of which is exponential decay and the other one is power-law decay. Hence, we fit the decay with three components as

1 $$I = A_1{\rm e}^{ - \frac{t}{{\tau _1}}} + A_2 {\rm e}^{ - \frac{t}{{\tau _2}}} + \frac{B}{{t + t_0}},$$

where *τ*_1_ and *τ*_2_ are related to the lifetimes of the slow and fast decay, respectively. *A*_1_, *A*_2_, *B* and *t*_0_ are other fitting parameters related to the initial population and rate constants. The value of *τ*_1_ is ~1 μs while *τ*_2_ has a value of ~10 ns.

For the small magnetic field case, instead of Eq. (1), a more complete model has to be used. In this more complete model, each valley has one WSe_2_ dark exciton state and two type of interlayer exciton states. The first type of the interlayer exciton decays following power law while the second type undergoes exponential decay. The dark exciton can scatter to the interlayer exciton of the second type. Additionally the dark exciton in one valley can scatter to become dark exciton in the other valley. This model is equivalent to Eq. (1) when this dark exciton intervalley scattering rate is negligible. The complete description of this model is given in the Supplementary Note 2 and Supplementary Fig. 3.

As can be seen from Fig. 4a, both the experimental data fits well with the theoretical model. The dark-to-interlayer exciton scattering rate is found to be in the MHz regime. This suggests that the dark exciton lifetime should be in the order of μs which is exceptionally long compared to the lifetime of other exciton types. The maximum value of the dark exciton intervalley scattering rate is found to be ~100 MHz at *B*_*z*_ = 0 T. It decreases quickly with increasing magnetic field. Note that this scattering rate is much smaller than the intralayer bright exciton intervalley scattering rate which can be attributed to the fact that the exchange interaction between dark excitons is much smaller compared to that between bright excitons. However, here we need to consider microsecond time scale. For this time scale, the valley depolarization of dark exciton need to be taken into account.

The dark-to-interlayer scattering rate has an exponential dependence on the magnetic field (Fig. 4d). This can be understood by analyzing the scattering mechanism from the dark exciton to the interlayer exciton. The dark exciton can scatter to become an interlayer exciton by following two different paths. These two paths are illustrated in Fig. 4b, c. In the first path (A-B-D path in Fig. 4b, c), the conduction band electron undergoes spin flipping before the charge transfer happens while in the second path (A-C-D path in Fig. 4b, c) the charge transfer happens before the spin flipping. Following the first path, the dark exciton will transform into an intermediate bright exciton before transforming into an interlayer exciton. According to previous measurement, charge transfer from the bright monolayer exciton to the interlayer exciton is very fast ~100 fs^7^. Therefore, we can safely neglect the charge transfer time. This means that the contribution of the first path to the dark-to-interlayer scattering rate will be approximately the same as the dark-to-bright exciton scattering rate. As can be seen from Fig. 4c this scattering rate at temperature *T* can be written as $r_0{\rm e}^{ - ({\mathrm{\Delta }}E_0 + g\mu _{\mathrm{B}})/k_{\mathrm{B}}T}$, where Δ*E*_0_ is the energy difference between the dark exciton and bright exciton at 0 T and *r*_0_ is the bright-to-dark exciton scattering rate. This shows that this contribution from the first path has an exponential dependence on magnetic field. On the contrary, the second path does not have strong magnetic field dependence because the transition only happens from a higher energy level to a lower energy level. Hence, its contribution to the dark-to-interlayer scattering rate is constant.

Based on our explanation above, the *g*-factor of the conduction band electron can be obtained from the magnetic dependence of the dark-to-interlayer exciton scattering rate at a fixed temperature. This can be used to check the sanity of our model. For K valley, the value 1.07 ± 0.079 is found while it is equal to   −1.11 ± 0.095 for K′ valley. These *g*-factor values agree well with the theoretical prediction of the conduction band *g*-factor for WSe_2_ when the out-of-plane effective spin *g*-factor has negative sign^45^. From the fitting in Fig. 4d, it can also be seen that the dark-to-interlayer exciton scattering rate saturates to a finite value of ~1 MHz at a big magnetic field as predicted by the model. Additionally, the value of the energy level difference between the bright and dark exciton at zero magnetic field (Δ*E*_0_) can also be calculated from the temperature dependence of *k*_1_. The detail of the experimental data and the theoretical fitting of the temperature dependence of *k*_1_ is shown in Supplementary Note 3 and Supplementary Fig. 4. The obtained energy level difference is in line with the value reported in ref. ^46^. All of these results shows the sanity of our model.

## Discussion

The fact that there are two types of interlayer exciton and that the time-resolved PL signal follows a multi-exponential decay has been reported before^2,30,31^. The component with slow exponential decay of this PL signal has been attributed to the extrinsic defect^30^ and also to a transition that is indirect in both real space and momentum space^31^. However, these two possible explanations cannot be used to explain the magnetic dependence of the slow decay rate that is observed in our data. Instead, we attribute this slow decay component to slow conversion rate from the dark exciton to the bright exciton. By doing so, the magnetic field dependence of this decay rate can be explained. Experimental demonstration of interlayer excitons on additional samples can be found in Supplementary Notes 5 and 6, Supplementary Figs. 7, 8 and 9.

In summary, we have experimentally demonstrated the long valley polarization lifetime in the order of microsecond in 2D heterostructures. This is primarily induced by magnetic field suppressed valley mixing for dark excitons. The long lifetime of the dark exciton put the dark exciton as a reservoir for the interlayer exciton in a long time scale. The long lifetime of exciton in 2D heterostructures makes 2D heterostructure to be a promising candidate for the realization of ultralong-distance exciton transport and exciton devices^13,14^. The possibilty to realize superfluidity^9^ in 2D heterostructures with a long exciton lifetime may provide future platform towards low-energy dissipation valleytronic devices.

## Methods

### Spectroscopy experiment setup

A homemade fiber-based confocal microscope is used for performing the polarization-resolved PL spectroscopy. Polarizers and quarter wave plates are installed on the excitation and detection arms of the confocal microscope for polarization-selective excitation and PL detection. The PL emission is directed by an multi-mode optical fiber into a spectrometer (Andor Shamrock) with a CCD detector for spectroscopic recording. The sample is loaded into a magneto cryostat and cooled down to ~2.3 K. Cryostat with vector magnet provides possibility to study dynamics in different magnetic field directions. The vector magnetic field ranges from −7 to +7 T in the out-of-plane direction (*z*-axis) and −1 to +1 T in the in-plane direction (*x*-axis and *y*-axis). The wavelength of the excitation is 726 nm (1.708 eV) for both the CW and pulsed laser experiment (pulse width 100 ps).

### Preparation of the heterostructures

We fabricated MoSe_2_/WSe_2_ heterostructures via a mechanical exfoliation and aligned-transfer method^7^. Bulk WSe_2_ and MoSe_2_ crystals (from HQ graphene) were used to produce WSe_2_ and MoSe_2_ monolayer flakes and they were precisely stacked with a solvent-free aligned-transfer process. We first prepared a WSe_2_ monolayer on SiO_2_ (300 nm)/Si substrate and a MoSe_2_ monolayer on a transparent polydimethylsiloxane (PDMS) substrate. After careful alignment (for both relative position and stacking angle) under the optical microscope with the aid of an XYZ manipulation stage, we then stacked the two monolayer flakes together, forming a PDMS/MoSe_2_/WSe_2_–SiO_2_/Si structure. Finally, we removed the top PDMS layer and obtained a MoSe_2_/WSe_2_ heterostructure on SiO_2_/Si substrate. For the controlled alignment of stacking angle, the armchair axes were guided according to their sharp edges from optical images, e.g., a stacking angle of 0° (60°) (<±2°) can be identified from Fig. 1a.

### Data availability

The data that support the findings of this study are available from the corresponding author upon request.

## Electronic supplementary material

Supplementary Information
