# Supplementary material for: Microsecond dark-exciton valley polarization memory in two-dimensional heterostructures
Source: Nat Commun. 2018 Feb 21;9:753. doi: 10.1038/s41467-018-03174-3 (PMC5821860; doi:10.1038/s41467-018-03174-3)
Supplement: Supplementary file 1 — Supplementary Information [file 41467_2018_3174_MOESM1_ESM.pdf]

Microsecond dark-exciton valley polarization memory in two-dimensional heterostructures

Chongyun Jiang,[1] Weigao Xu,[1] Abdullah Rasmita,[1] Zumeng Huang,[1] Ke Li,[1] Qihua Xiong,[1,2,3] and Wei-bo Gao[1,3,4]

[1]Division of Physics and Applied Physics, School of Physical and Mathematical Sciences, Nanyang Technological University, Singapore 637371, Singapore

[2]NOVITAS, Nanoelectronics Center of Excellence, School of Electrical and Electronic Engineering, Nanyang Technological University, 639798 Singapore

[3]MajuLab, CNRS-Université de Nice-NUS-NTU International Joint Research Unit UMI 3654, Singapore

[4]The Photonics Institute and Centre for Disruptive Photonic Technologies, Nanyang Technological University, 637371 Singapore, Singapore

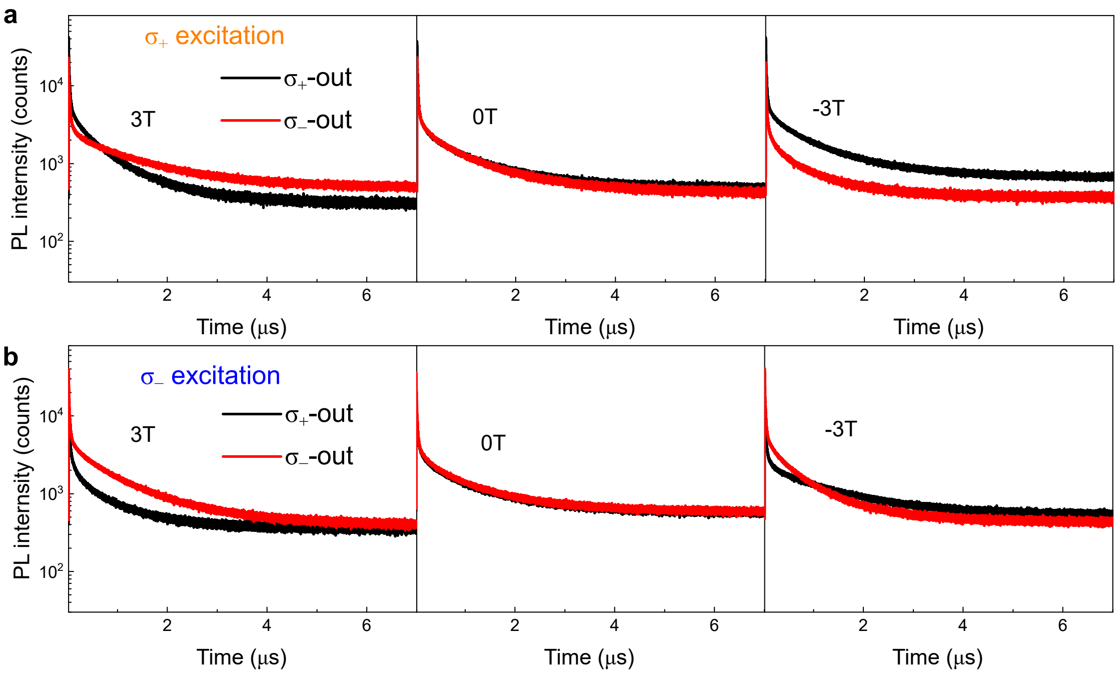

Supplementary Figure 1: Time-resolved PL. a, The three panels represent the time-resolved PL under σ_+ excitation at out-of-plane magnetic field B_z =+3 T, 0 T and -3 T. **b**, similar as **a** but under σ_- excitation.

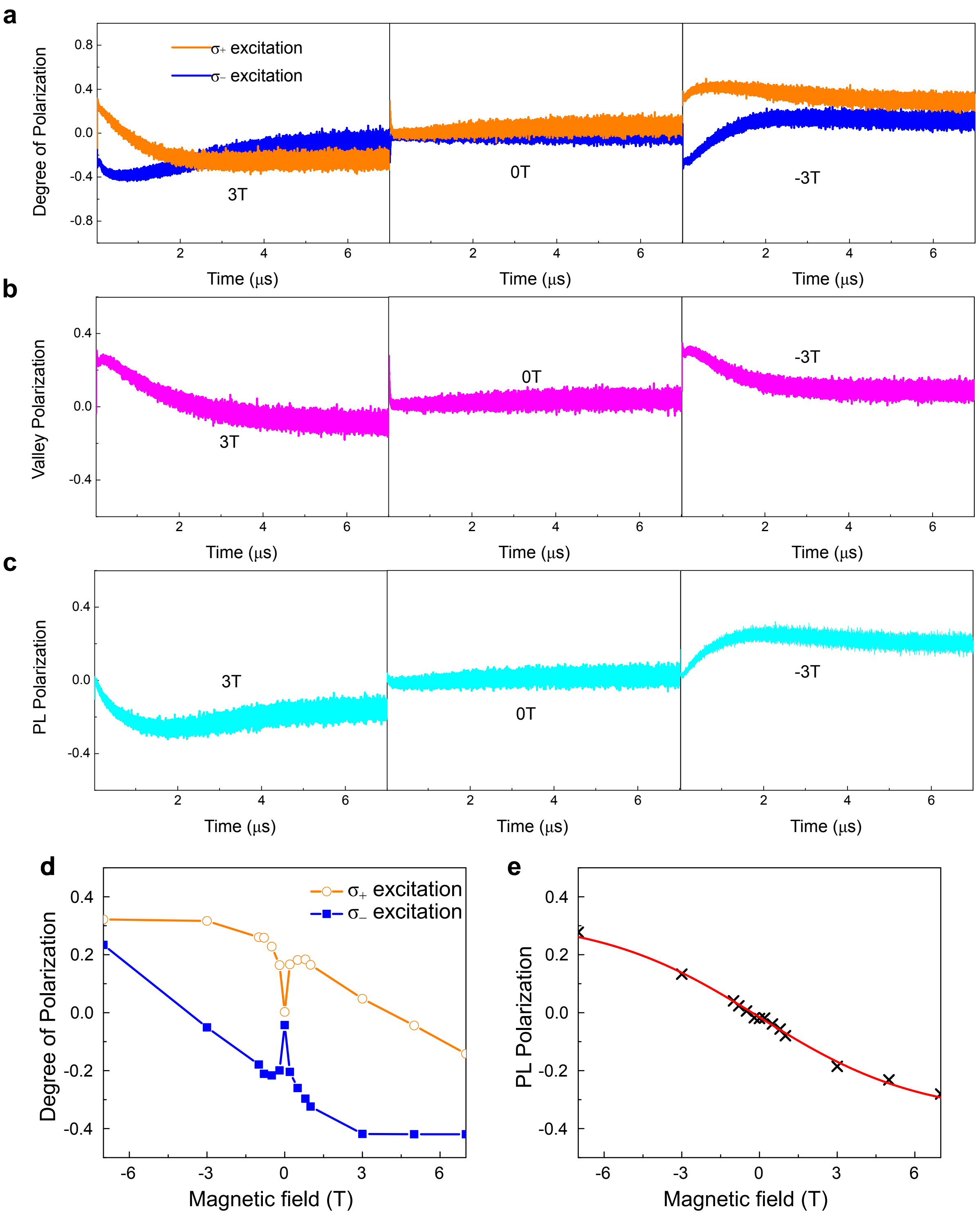

Supplementary Figure 2: **Time-resolved degree of polarization**. **a**, The degree of polarization P^j for σ_+ excitation at out-of-plane magnetic field B_z =+3 T, 0 T and -3 T. Here $P^j = \frac{I^j_{\sigma_+} - I^j_{\sigma_-}}{I^j_{\sigma_+} + I^j_{\sigma_-}}$,where j indicates the excitation polarization and $I^j_{\sigma_+}$ ($I^j_{\sigma_-}$) is the $\sigma_+(\sigma_-)$ polarized PL intensity when excitation with j polarization is used. **b**, Valley polarization $P_{val} = \frac{P^{\sigma_+} - P^{\sigma_-}}{2}$ at out-of-plane magnetic field B_z =+3 T, 0 T and -3 T. **c**, PL polarization $P_{\mathrm{PL}} = \frac{P^{\sigma_+} + P^{\sigma_-}}{2}$ at out-of-plane magnetic field B_z =+3 T, 0 T and -3 T. **d**, The degree of polarization as a function of B_z with pulsed laser excitation. The intensity is integrated from 21 ns to 7 μs. **e**, The PL polarization as a function of B_z with pulsed laser excitation. The PL polarization shows linear dependence on magnetic field for small magnetic field before saturating at big magnetic field.

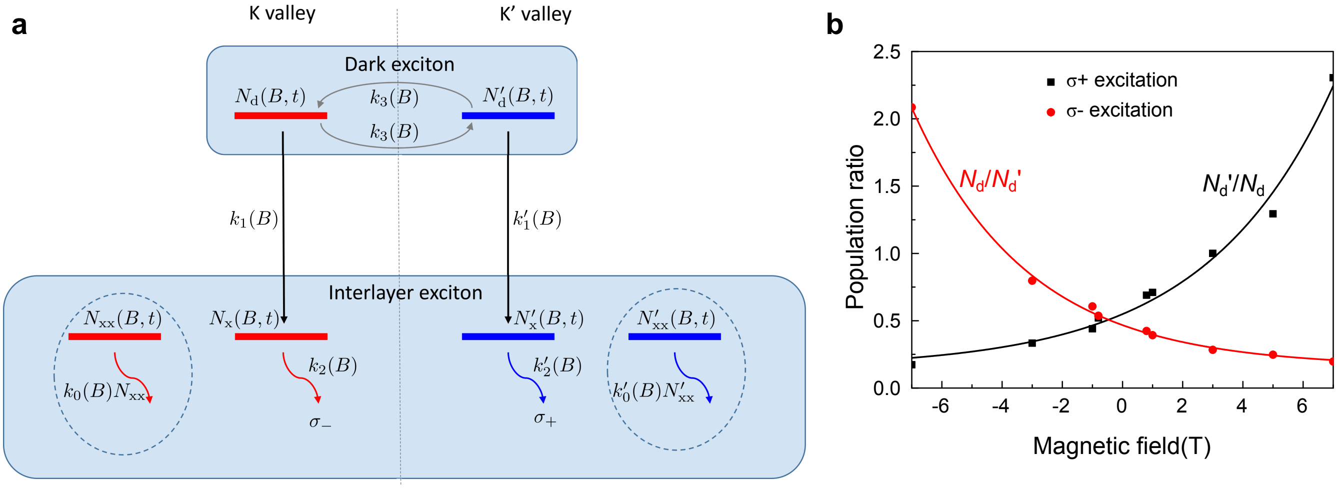

Supplementary Figure 3: **Theoretical model and fitting parameters**. **a**, Model description. The term $N_{\mathrm{d}}(N'_{\mathrm{d}})$, and $N_{\mathrm{x}}(N'_{\mathrm{x}})$, are the population of K (K') valley's dark exciton and interlayer exciton while $N_{\mathrm{xx}}(N'_{\mathrm{xx}})$ corresponds to the population of the additional exciton level at K (K') valley to account for the power decay with the decay rate equal to $k_0 N_{\mathrm{xx}}$ for K valley and $k'_0 N'_{\mathrm{xx}}$ for K' valley. The term k_3 is the intervalley scattering rate, $k_1(k'_1)$ is the dark-to-interlayer scattering rate and $k_2(k'_2)$ is the interlayer exciton decay rate. The term B and t denote the magnetic field and time dependence of the parameters. **b**, The ratio between the dark exciton population at K and K' valley at different excitation polarization. The population ratio shows exponential dependence on magnetic field.

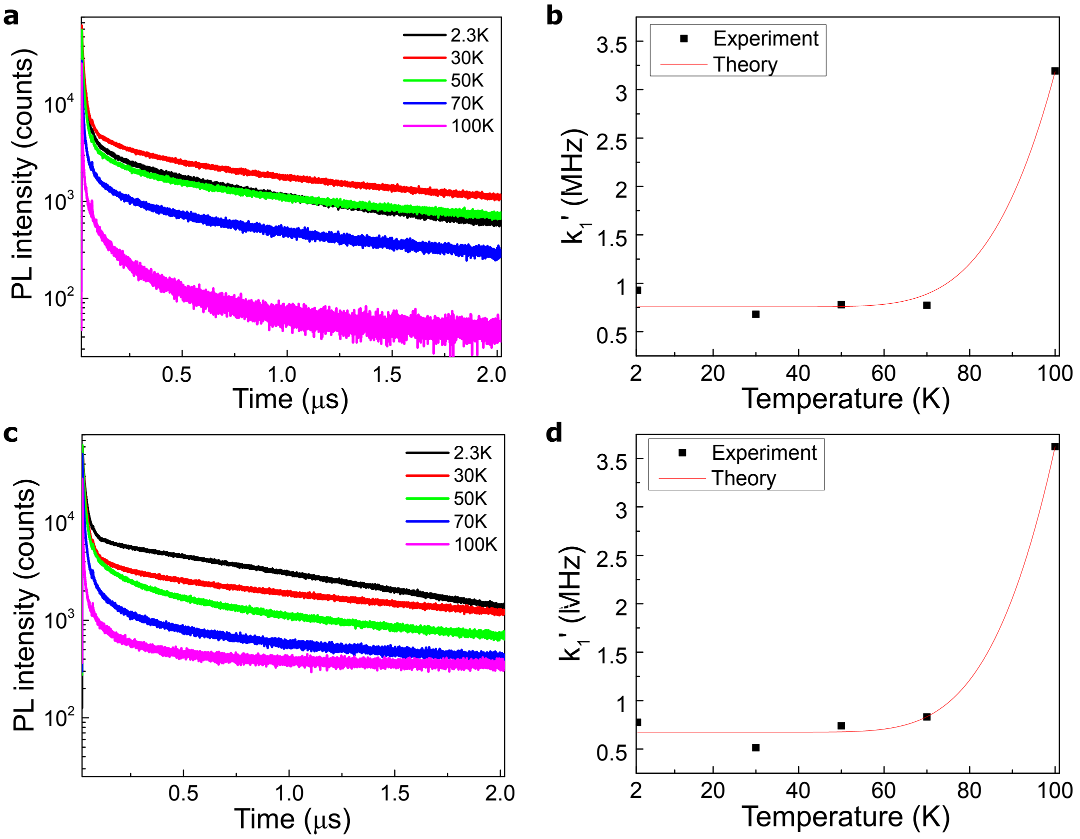

Supplementary Figure 4: **Temperature dependence of the interlayer exciton PL**. The σ_- polarized PL at $B = 0$ T and $B = 7$ T are shown in **a** and **c** respectively. In both case, σ_- polarized pulsed excitation were used. The temperature dependence of the corresponding dark-to-interlayer exciton scattering rate(k_1) is shown in **b** and **d** respectively.

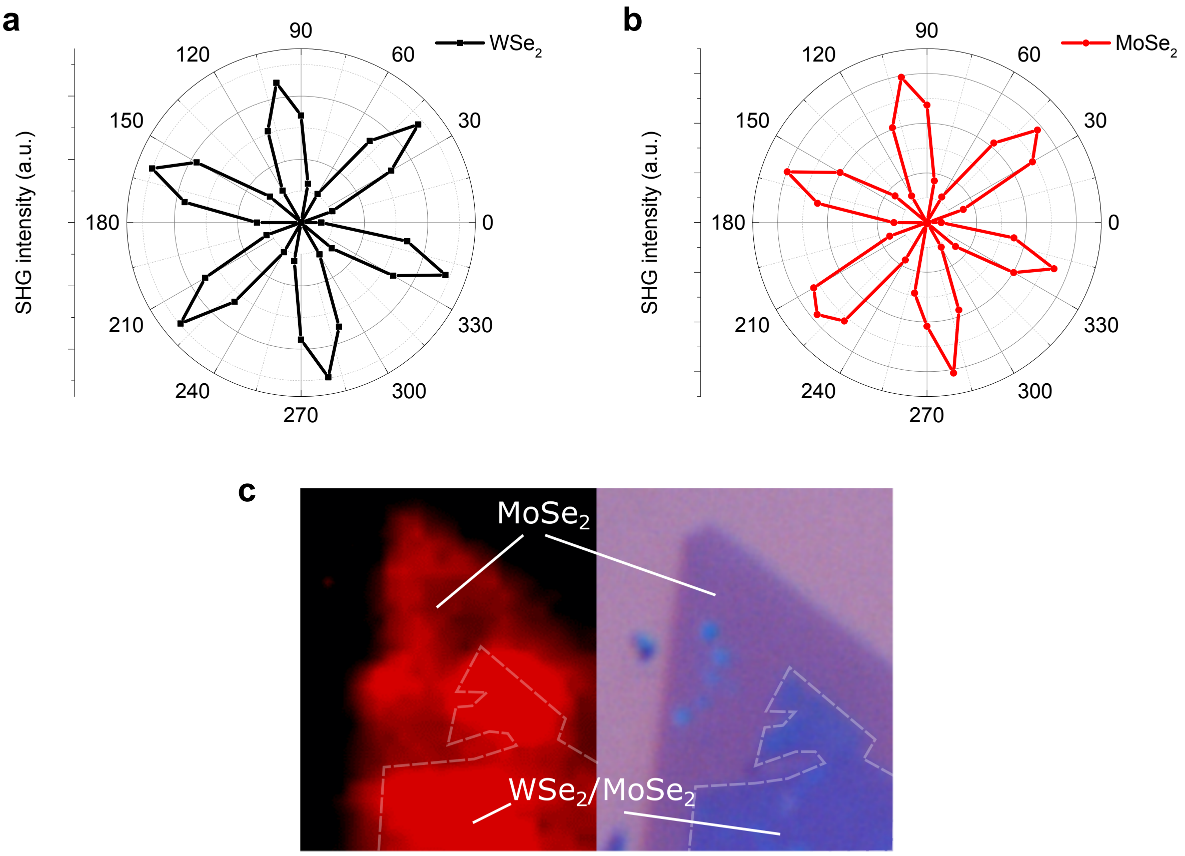

Supplementary Figure 5: **Stacking sequence determination using second harmonic generation (SHG) measurement.** SHG signal from monolayer WSe_2 (**a**) and $MoSe_2$ (**b**) of one of the $WSe_2/MoSe_2$ heterostructure samples. **c**, SHG mapping. The heterostructure shows a stronger SHG than that of the individual monolayers, which implies that the staking angle of the two monolayers is 0 degree, i.e. AA-staking.

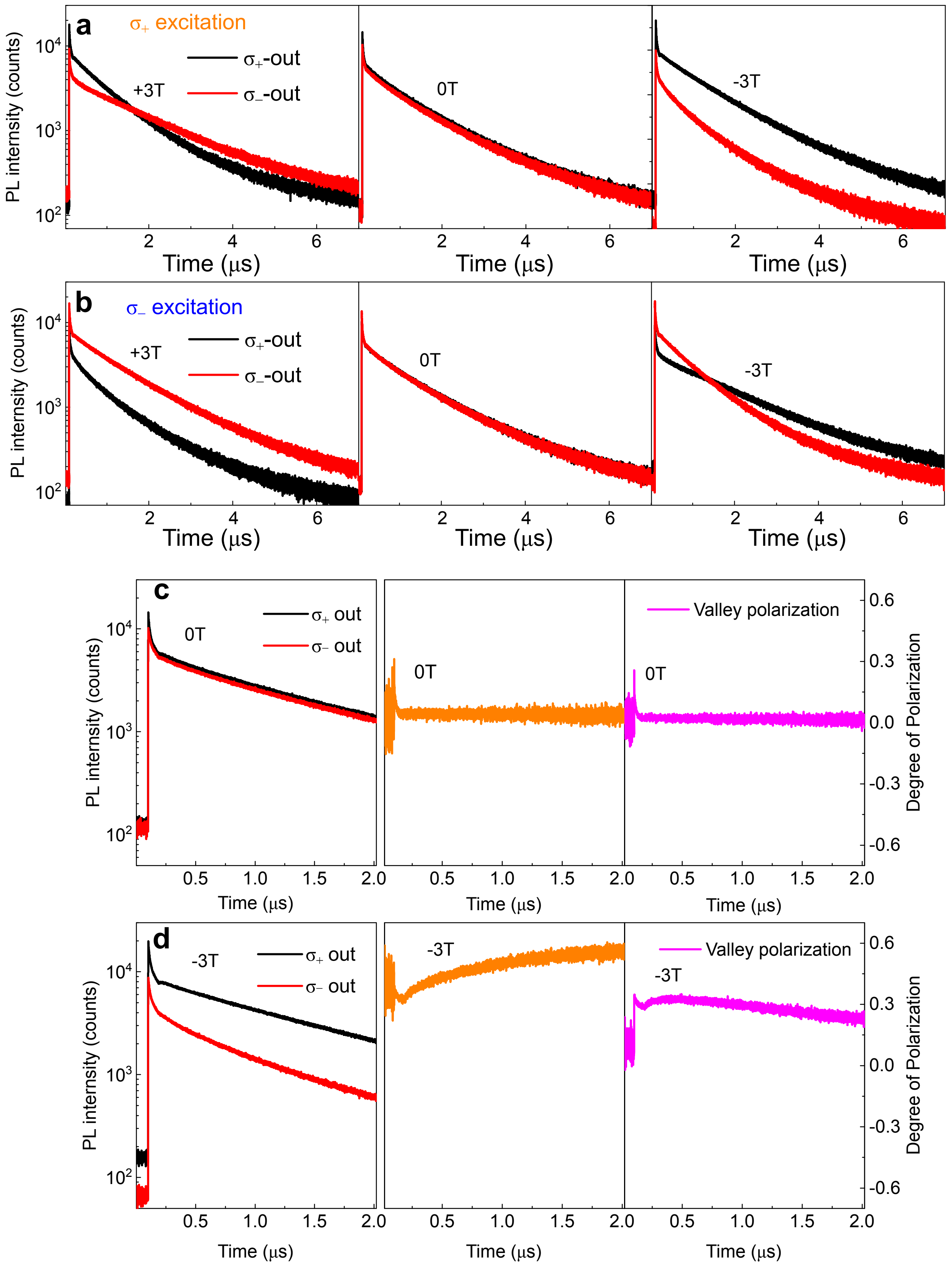

Supplementary Figure 6: **Time resolved PL (second** $\mathbf{MoSe_2/WSe_2}$ **sample).** **a**, Time resolved PL under σ_+ polarized 726 nm pulsed laser excitation. The three panels represent the PL at out-of-plane magnetic field +3 T (left panel), 0T (middle panel) and -3 T (right panel). **b**, Similar as **a**, but under σ_- polarized excitation. **c**, The left panel shows the decay of the PL emission while the middle and the right panel shows the calculated degree of polarization and valley polarization respectively. **d**, Similar as **c**, but under -3 T out-of-plane magnetic field.

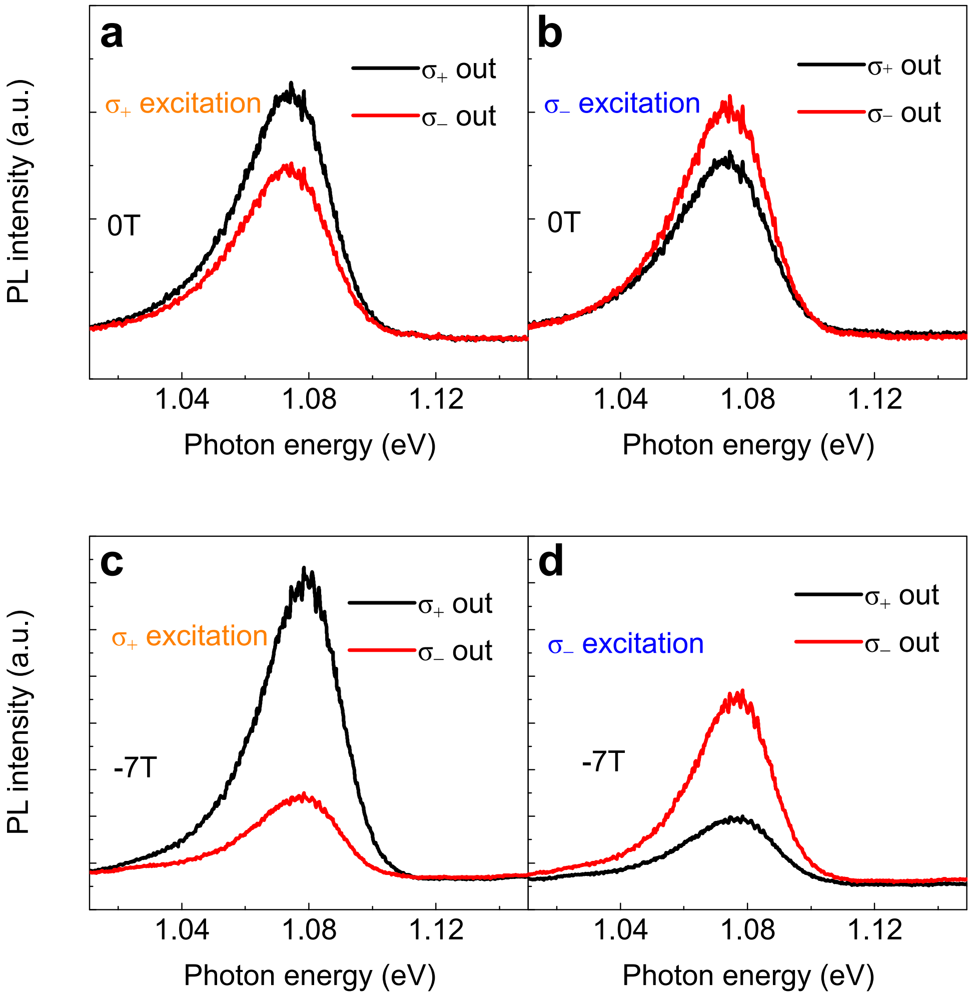

Supplementary Figure 7: MoS_2/WSe_2 sample PL spectra. PL spectra under 726 nm CW excitation and out-of-plane magnetic field 0 T (**a**, **b**) and -7 T (c,d).

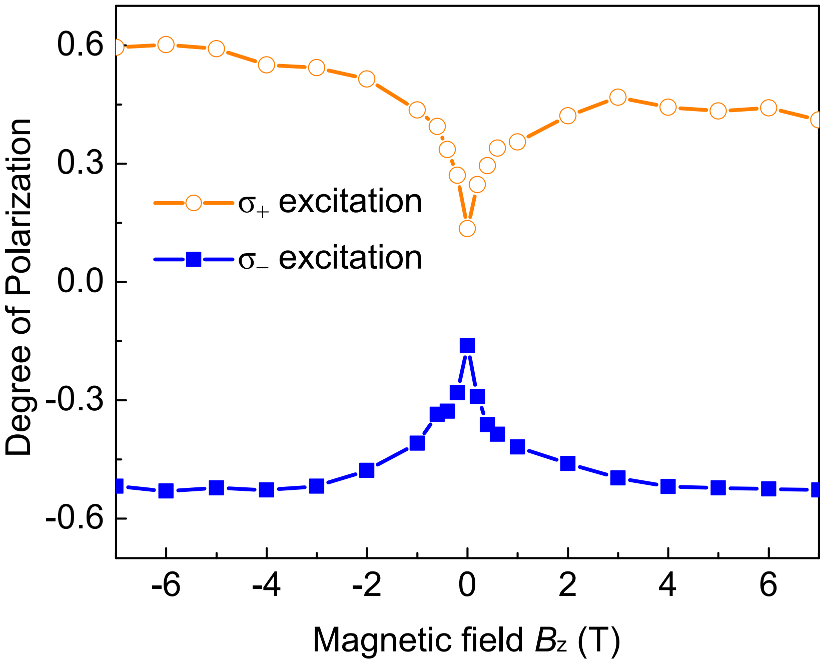

Supplementary Figure 8: **MoS_2/WSe_2 degree of polarization.** The degree of polarization of the MoS_2/WSe_2 sample is plotted as a function of out-of-plane magnetic field under 726 nm CW excitation.

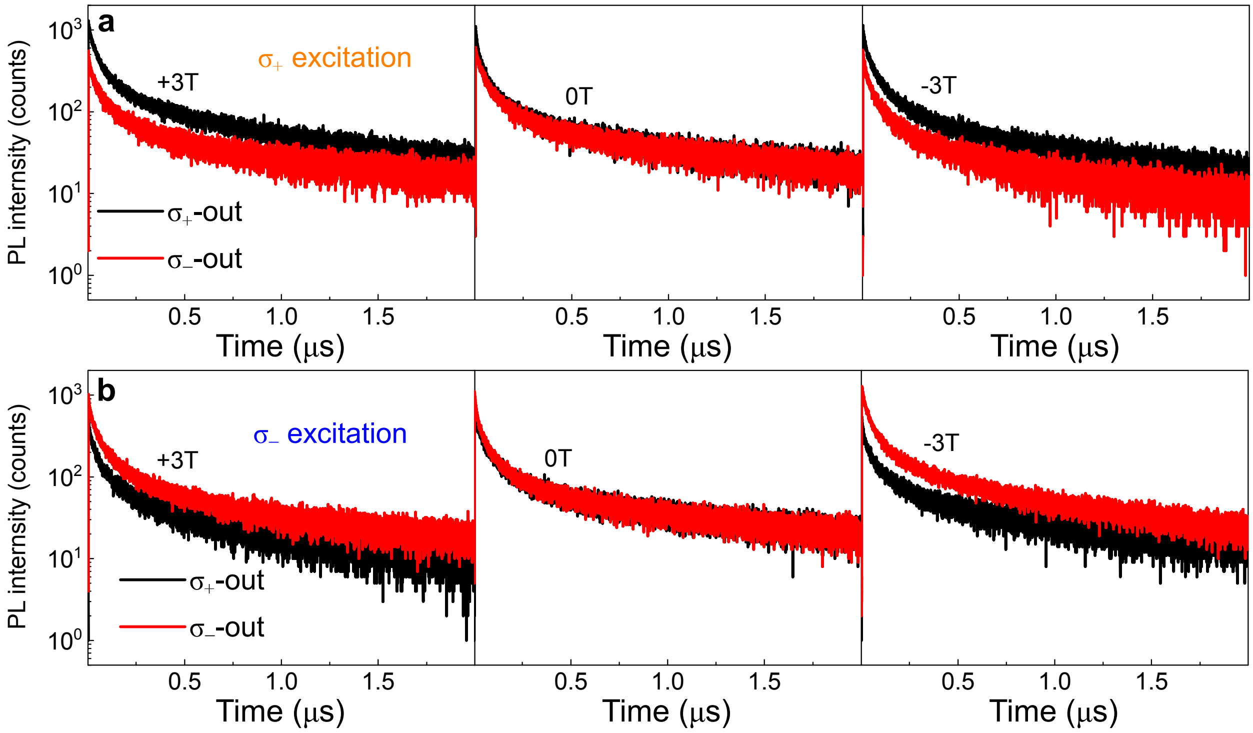

Supplementary Figure 9: **MoS_2/WSe_2 time-resolved PL. a**, Time resolved PL under σ_+ polarized 726 nm pulsed laser excitation. The three panels represent the PL at out-of-plane magnetic field +3 T (left panel), 0T (middle panel) and -3 T (right panel). **b**, Similar as **a**, but under σ_- polarized excitation.

Supplementary Note 1. ANALYSIS OF TIME-RESOLVED DEGREE OF POLARIZATION

The time resolved interlayer PL emission for $B = 3, 0, -3$ T for σ_+ and σ_- are shown in Supplementary Figure 1a and 1b respectively. Based on this measurement, the time resolved degree of polarization can be determined. The time-resolved degree of polarization, the valley polarization and the PL polarization are shown in Supplementary Figure 2a, 2b, and 2c respectively.

As can be seen from Supplementary Figure 2, the lifetime of the valley polarization at 3 T and -3 T are similar to each other. In both cases, the valley polarization lifetime is much bigger than the 0T case. As has been explained in the main text, this is due to the suppression of the intervalley scattering when an out-of-plane magnetic field is applied. This is not the case for the PL polarization. The PL polarization at -3 T differs from the one at 3 T. In particular it saturates to different values at different magnetic field: ~ 0.2 at -3 T, and ~ -0.2 at 3 T. This can be understood by the following scenario.

Initially, the polarized pulsed laser excites the bright exciton (trion) in one valley. This bright exciton population undergoes 4 main processes. It can: **(1)** relax to vacuum which results in intralayer emission, **(2)** transform to interlayer exciton through charge transfer between $MoSe_2$ and WSe_2, **(3)** undergo intervalley scattering to the bright exciton in different valley, and **(4)** transform to dark exciton through the spin flipping of the conduction band electron.

The first three processes happen in short time scale and they together determine the initial population of the interlayer exciton, which decays in tens of nanoseconds. This population decay is responsible for the fast decay of the valley polarization as shown in the inset of the main text Figure 3e right panel. In the later stage (> 50 ns), the dark exciton contribution to the interlayer exciton emission is the dominant process. In other words, the valley polarization and PL polarization of the interlayer exciton closely follow the dark exciton population decay dynamics. At more than 6 μs, since the thermal equilibrium has been reached, the degree of polarization has a weak dependence on the excitation polarization and it is determined mostly by the dark exciton population in the two valleys which follows Boltzmann distribution. Hence, the valley polarization saturates to a near zero value and the PL polarization saturates to a value determined by the dark exciton distribution between the two valleys which depends on the magnetic field.

Additionally, we can integrate the PL signal to get the total emission from each valley and

plot the degree of polarization and PL polarization of this total emission as a function of out-of-plane magnetic field. This is shown in Supplementary Figure 2d and 2e respectively. As shown in Supplementary Figure 2d, the degree of polarization becomes larger when we apply a small magnetic field. This is similar with the CW excitation case. However, it becomes asymmetric for larger magnetic field. It increases to a saturation level for one magnetic field direction while it does not show saturation behavior for the opposite direction. This non-saturation behavior is mainly caused by the increased PL polarization, P_{PL}, in the long timescale at a large magnetic field. The plot of the P_{PL} against out-of-plane magnetic field is shown in Supplementary Figure 2e.

The energy degeneracy break down of the dark excitons in different valley is the primary reason for the magnetic field dependence of the observed PL polarization. At a finite magnetic field, the population ratio between these energy levels at thermal equilibrium is determined by the Boltzmann distribution. Because of this, the PL polarization will have a near-linear magnetic field dependence at small magnetic field and saturate to the value of 1 at big magnetic field. However, due to the valley polarization, the magnitude of the PL polarization will be less than 1. Following this line of reasoning, the PL polarization is fitted using equation $P_{\mathrm{PL}} = P^{s}_{\mathrm{PL}} \frac{1-e^{-\beta B}}{1+e^{-\beta B}} + P^{0}_{\mathrm{PL}}$, where $\beta = \frac{g_{\mathrm{v}} \mu_{\mathrm{B}} B}{k_{\mathrm{B}} T}$ with g_{v} as the effective g-factor between K and K' valley, P^{s}_{PL} is the saturation level of the PL polarization, and P^{0}_{PL} is a small residual PL polarization at 0 T which is most probably caused by experimental imperfections. The result of this fitting is shown in Supplementary Figure 2e. Based on the fitting result, we found the value of $g_{\mathrm{v}} = 1.1 \pm 0.09$ for the effective g-factor between the valleys.

Supplementary Note 2. DETAIL OF THEORETICAL MODEL AND EXPERIMENTAL DATA FITTING

The diagram illustrating the theoretical model showing all the parameters involved in the fitting are shown in Supplementary Figure 3a. The interlayer exciton emission is proportional to the sum of the two interlayer exciton population. That is $I_{\sigma+} \propto (N_{\mathrm{xx}} + N_{\mathrm{x}})$ and $I_{\sigma-} \propto (N'_{\mathrm{xx}} + N'_{\mathrm{x}})$.

The rate equation model can be solved analytically. The closed form solutions for N_{d}, N'_{d}, N_{x}, N_{x}', N_{xx} and N'_{xx} are

$$N_{\mathrm{d}} = c_1 e^{-tk_-} + c_2 e^{-tk_+} \quad (1)$$

$$N_{\mathrm{x}} = c_3 e^{-tk_2} + c_1 \frac{k_1}{k_2 - k_-} e^{-tk_-} - c_2 \frac{k_1}{k_+ - k_2} e^{-tk_+} \tag{2}$$

$$N_{\mathrm{xx}} = \frac{1/k_0}{t + c_4} \tag{3}$$

$$N'_{\mathrm{d}} = c_1 \frac{k_3}{\sqrt{\Delta^2 + k_3^2} - \Delta} e^{-tk_-} - c_2 \frac{k_3}{\sqrt{\Delta^2 + k_3^2} + \Delta} e^{-tk_+} \tag{4}$$

$$N'_{\mathrm{x}} = c_5 e^{-tk'_2} + c_1 \frac{k_3}{\sqrt{\Delta^2 + k_3^2} - \Delta} \frac{k'_1}{k'_2 - k_-} e^{-tk_-} + c_2 \frac{k_3}{\sqrt{\Delta^2 + k_3^2} + \Delta} \frac{k'_1}{k_+ - k'_2} e^{-tk_+} \tag{5}$$

$$N'_{\mathrm{xx}} = \frac{1/k'_0}{t + c_6}. \tag{6}$$

where $k_\pm = \frac{k_1 + k'_1}{2} + k_3 \pm \sqrt{\Delta^2 + k_3^2}$ and $\Delta = \frac{k_1 - k'_1}{2}$. The terms $N_{\mathrm{d}}(N'_{\mathrm{d}})$, and $N_{\mathrm{x}}(N'_{\mathrm{x}})$, are the population of K (K') valley's dark exciton and interlayer exciton. The term $N_{\mathrm{xx}}(N'_{\mathrm{xx}})$ corresponds to the population of the additional exciton level at K (K') valley to account for the power decay with decay rate equal to $k_0 N_{\mathrm{xx}}$ for K valley and $k'_0 N'_{\mathrm{xx}}$ for K' valley. k_3 is the intervalley scattering rate, $k_1(k'_1)$ is the dark-to-interlayer scattering rate and $k_2(k'_2)$ is the interlayer exciton decay rate. All c_i terms are constants that depend on the initial condition.

It is possible to fit these equations straight away to the experimental data. However, due to the large number of the independent variables, there is a possibility that multiple local optimum solutions exist with some of the solution is not physically reliable. A different strategy can be employed by using the fact that for magnetic field with big enough magnitude the value of k_3 is negligible. In our case, we assume $k_3 = 0$ MHz for $|B| = 0.8$ T, which is reasonable given the saturation of valley polarization happens at $|B| > 0.8$ T. In this case the interlayer population $N_{\mathrm{x}}(N'_{\mathrm{x}})$ and the dark exciton population $N_{\mathrm{d}}(N'_{\mathrm{d}})$ can be approximated as

$$N_{\mathrm{d}} = c_2 e^{-tk_1} \tag{7}$$

$$N_{\mathrm{x}} = c_3 e^{-tk_2} + c_2 \frac{k_1}{k_2 - k_1} e^{-tk_1} \tag{8}$$

$$N'_{\mathrm{d}} = c_7 e^{-tk'_1} \tag{9}$$

$$N'_{\mathrm{x}} = c_5 e^{-tk'_2} + c_7 \frac{k'_1}{k'_2 - k'_1} e^{-tk'_1}. \tag{10}$$

As can be seen from Supplementary Equation 7-10, for big magnetic field, there is no coupling between the exciton population in different valley. Hence, in this case, the model is equivalent to a time-dependent function with two exponentials and one power decay as stated in the main text. Moreover, due to the absence of the coupling terms, the experimental PL data for different emission polarization can be fitted separately.

The fitting result at big magnetic field can be used to check the sanity of the model. The model predicts that the value of various decay rates (k_1, k_1', k_2, k_2', k_0 and k_0') and the population ratio between the interlayer exciton and dark exciton in one valley should be independent of the excitation polarization. We found that this is the case for all of these parameters other than the small difference for power decay rate (k_0 and k_0').

From the fitting result at big magnetic field, we extract the magnetic field dependence of $k_1, k_1', k_2, k_2', \frac{N_\mathrm{d}}{N_\mathrm{x}+N_\mathrm{xx}}, \frac{N_\mathrm{d}'}{N_\mathrm{x}'+N_\mathrm{xx}'}$, and $\frac{N_\mathrm{d}+N_\mathrm{x}+N_\mathrm{xx}}{N_\mathrm{d}'+N_\mathrm{x}'+N_\mathrm{xx}'}$. From these data, the values of these parameters at small magnetic field are interpolated by using linear interpolation. The value of k_0, k_0', N_xx, and N_xx' are obtained by doing the fitting using Supplementary Equation 7-10 for both small and big magnetic field. The intervalley scattering rate (k_3) is used as a fitting parameter in Supplementary Equation 1-6 to fit the PL data for the case of small magnetic field. As shown in Figure 4a in the main text, the fitting result fits well with the experimental data.

The ratio between the dark exciton population in K and K' valley obtained from this fitting (see Supplementary Figure 3b) can be used to get the dark exciton g-factor. Magnetic field will lift the energy degeneracy of dark excitons and cause the population difference between the two valleys. Based on this fitting, we find this g factor to be equal to 0.9 ± 0.04. This is quite close to the value of effective g-factor between K and K' (see Supplementary Note 1), which indicates that dark exciton dynamics dominates the total PL polarization.

We note that our rate equation model is similar to the rate equation model in [1] where it is used very well to explain the increase of valley polarization with increasing magnetic field in WSe_2 local exciton case. However, we found that this model has to be extended in order to explain our finding. Unlike in [1] where the scattering rate between the dark exciton and the local exciton is treated as a constant, in our case the scattering rate between the dark exciton and interlayer exciton (k_1 and k_1) depends on the magnetic field and the valley.

Supplementary Note 3. TEMPERATURE DEPENDENCE OF DARK-TO-INTERLAYER EXCITON SCATTERING RATE

In order to get the value of the energy level difference between bright and dark exciton at zero magnetic field (ΔE_0), the pulse measurement were done in various temperatures at two different magnetic field settings: $B = 0$ T and $B = 7$ T. These data is then fitted with the theoretical model to obtain the value of dark-to-interlayer exciton scattering rate (k_1).

The measurement results for the case where σ_- polarized pulsed excitation and σ_- polarized PL detection are used can be seen in Supplementary Figure 4a (for $B = 0$ T) and 4c (for $B = 7$ T). The corresponding temperature dependence of k_1 at two different magnetic field settings is shown in Supplementary Figure 4b (for $B = 0$ T) and 4d (for $B = 7$ T). Based on this fitting, we obtain $\Delta E_0 = 58.2 \pm 20$ meV. Considering the experimental uncertainty, this result is comparable to the value of this parameter reported in [2] and [3].

Supplementary Note 4. STACKING SEQUENCE OF THE SAMPLE

It is possible to determine the stacking sequence of the bilayer heterostructure using second harmonic generation (SHG) measurement [4]. If the two monolayers are 0-degree aligned (AA stacking), the SHG emission from the two monolayers will add together and therefore the SHG signal in the heterostructure area should be larger compared to the monolayer SHG signal. For 60-degree alignment (AB stacking), the SHG signal in the heterostructure is lower than that in monolayers because of destructive interference between the SHG signals from different layer. We have implemented this SHG measurement. The angle dependent SHG for WSe_2 and $MoSe_2$ and the SHG for $WSe_2/MoSe_2$ heterostructure are shown in Supplementary Figure 5a, 5b, and 5c respectively. We found that the SHG signal from the heterostructure is larger than that from the individual $MoSe_2$ and WSe_2 monolayer. Based on this measurement result, we conclude that the stacking sequence of our heterostructure is AA stacking.

Supplementary Note 5. DATA FROM ADDITIONAL $MoSe_2/WSe_2$ SAMPLE

In this section we presents the measurement result of one additional $MoSe_2/WSe_2$ sample (see Supplementary Figure 6). In all of our experiments, we observed that applying an out-of-plane

magnetic field increases the valley polarization. The pulse experiment result across various samples shows similar behavior as the result reported in the main text.

Supplementary Note 6. DATA FROM MoS_2/WSe_2 SAMPLE

The conclusion that we derived from our $MoSe_2/WSe_2$ experimental result can be extended to other similar heterostructure systems such as MoS_2/WSe_2 heterostructures. In order to show this, we presents the measurement result of one MoS_2/WSe_2 sample (Supplementary Figure 7-9). It is worth to mention that the wavelength for interlayer exciton in the MoS_2/WSe_2 heterostructure is ~1150 nm. Based on our knowledge, this is the first report of interlayer exciton at this frequency range.

Supplementary References

[1] Smoleński, T. *et al.* Tuning valley polarization in a WSe_2 monolayer with a tiny magnetic field. *Phys. Rev. X* **6**, 021024 (2016).

[2] Molas, M. R. *et al.* Brightening of dark excitons in monolayers of semiconducting transition metal dichalcogenides. *2D Materials* **4** (2017).

[3] Zhang, X.-X. *et al.* Experimental evidence for dark excitons in monolayer WSe_2. *Phys. Rev. Lett.* **115**, 257403 (2015).

[4] Hsu, W.-T. *et al.* Second harmonic generation from artificially stacked transition metal dichalcogenide twisted bilayers. *ACS Nano* **8 (3)**, 2951–2958 (2014).
